# Stigma, disfigurement and resilience among acid attack survivors: a qualitative body mapping study in Noida, India

**DOI:** 10.1136/bmjph-2025-002693

**Published:** 2026-01-27

**Authors:** Pratishtha Singh, Vikash Ranjan Keshri, Ridhima Rathi, Jagnoor Jagnoor

**Affiliations:** 1 Injury, The George Institute for Global Health India, New Delhi, India; 2 Faculty of Medicine and Health, University of New South Wales, Sydney, New South Wales, Australia; 3 Jindal School of Public Health and Human Development, O P Jindal Global University, Sonipat, India; 4 Independent Consultant, New Delhi, India; 5 Injury, The George Institute for Global Health, Sydney, New South Wales, Australia

**Keywords:** Female, Public Health, Social Medicine, Qualitative Research, Violence

## Abstract

**Background:**

Burn injuries are a significant global public health concern, particularly in low- and middle-income countries like India. Acid attack is a type of intentional chemical burn and a form of gender-based violence used to disfigure the victim. While the physical and psychological impacts of acid attacks are documented, research focusing on the stigma experienced by survivors in India is limited. This study aims to explore how acid attack survivors in India experience and negotiate stigma associated with disfigurement.

**Methods:**

This qualitative research was conducted between November 2023 and April 2024 in Noida, India, in collaboration with the Chhanv Foundation—a community-based non-governmental organisation that works to support acid attack survivors. The study used body mapping, a participatory art-based methodology with eight women survivors of acid-attack violence. The body mapping exercise was conducted over four face-to-face sessions, ranging from 3 to 6 hours each. Two focus group discussions were also conducted. Thematic analysis was done in NVivo.

**Results:**

Two meta-themes were identified as factors contributing to stigma and factors mitigating stigma. The first theme highlights the devaluation of bodies with disfigurement, unacceptance and abuse from family, and the resulting loss of educational and employment opportunities. The second theme of factors mitigating stigma documents participants’ journey towards self-acceptance and the critical role of the non-governmental organisation in providing opportunities and engaging them in advocacy.

**Conclusions:**

This is the first study globally to use body mapping with acid attack survivors. The study findings recommend integrating holistic rehabilitation programmes that include psychosocial support, education and advocacy. It also illustrates the value of arts-based participatory research methods in capturing the lived experiences of survivors, ultimately contributing to their empowerment.

**WHAT IS ALREADY KNOWN ON THIS TOPIC**

- Acid violence is a severe form of gender-based violence, disproportionately affecting women and girls, with long-term physical, psychological and social consequences. Existing research is largely anecdotal, addressing the legal aspects of acid attacks.

**WHAT THIS STUDY ADDS**

- We qualitatively documented narratives of stigmatisation by acid attack survivors in Noida, India. Using body mapping and focus group discussions, we provided a platform for survivors to articulate their experiences around stigma in a multidimensional and participatory manner. We found that stigma is shaped by contributing factors such as familial rejection, societal devaluation and opportunity loss, while mitigating factors like non-governmental organisation support and self-acceptance play a critical role in resilience and recovery.

**HOW THIS STUDY MIGHT AFFECT RESEARCH, PRACTICE OR POLICY**

- This study highlights the urgent need for holistic rehabilitation policies that address not just the medical but also the psychosocial and economic needs of acid attack survivors. By demonstrating the value of body mapping as a participatory method, it also encourages future research to adopt arts-based methodologies to better capture lived experiences and inform survivor-centred interventions.

## Introduction

 Burn injuries are a significant global public health concern, with the World Health Organization (WHO) estimating around 180 000 deaths annually.^1^ A specific type of intentional burn injury is acid violence, in which corrosive chemical substances are used to cause disfigurement, injury or death, resulting in acid attack or ‘vitriolage.’ These attacks are a form of gender-based violence, disproportionately affecting women and girls, often motivated by an intention to severely disfigure the victim.^2^ The majority of cases occur in low-income and middle-income countries (LMICs), with data from the Acid Survivors Trust International reporting over 200 acid attack cases every year in India.^3^ However, the actual incidence is expected to be higher due to underreporting and inconsistency in data, with estimates suggesting more than 1000 cases annually.^4 5^ Nationally representative survey data further indicate a 1% prevalence of severe partner-perpetrated burns in India, with social correlates including in-law violence and younger age at marriage, while greater wealth and having a son appear protective.^6^

The impact of acid attacks is multifaceted, affecting survivors’ physical health, psychological well-being and social integration.^27^,^10^ Survivors often face extensive and costly reconstructive surgeries and long-term medical treatments, which can lead to significant functional impairments and an overall low quality of life.^3^ They also encounter social stigma and exclusion, which can exacerbate challenges.^7^ Stigma, a major social determinant of health, is described as the ‘hidden’ burden of disease and comprises cognitive, emotional and behavioural components.^11^,^13^ Perceived stigma involves understanding how others may act towards individuals based on certain traits or identities, while anticipated stigma refers to expectations of future stigma experiences.^14 15^ Internalised stigma refers to the individual-level process of acknowledging, accepting and applying stigma to oneself, with experienced or enacted stigma involving instances of discriminatory acts or behaviours.^14 16^

Theorists such as Link and Phelan^11^ have emphasised that stigma is not simply an individual experience but a social process embedded within power structures. Their model outlines interconnected mechanisms of labelling, stereotyping, separation, and status loss, all occurring within unequal social relations that reinforce marginalisation. In the context of disfigurement, theories of visible difference suggest that stigma is intensified by the body’s visibility as a site of social meaning and moral judgement.^17^ Disfigurement challenges dominant beauty norms and social expectations, often leading to appearance anxiety and social avoidance.^18^ These frameworks together shape our understanding of acid attack stigma as a dynamic process operating across structural, social and embodied dimensions.

Despite the severe implications for survivors’ well-being, research on the long-term outcomes of acid attacks in India is negligible. Existing literature is largely anecdotal, addressing the legal aspects of acid violence,^419^,^22^ with insufficient attention to the stigma experienced by survivors. This study aims to address this gap by exploring the lived experiences of acid attack survivors in India, with a particular focus on the stigma associated with disfigurement, using body mapping methodology. Our study was guided by the following research question ‘How do acid attack survivors in India experience and negotiate stigma associated with disfigurement?’ To answer this, we used the following subquestions:

1. What social, familial, and structural factors contribute to the stigmatisation of acid attack survivors?
2. What individual and collective mechanisms support resilience, self-acceptance, and recovery from stigma?

## Materials and methods

We conducted a qualitative inquiry to document factors associated with the stigma faced by acid violence survivors using body mapping and focus group discussions (FGDs) in Noida, Uttar Pradesh, India. It was conducted in collaboration with the Chhanv Foundation, a non-governmental organisation (NGO). No patients or individuals from the public were involved in the design, or conduct, or reporting, or dissemination plans of our research.

### Context

Chhanv Foundation, an NGO based out of Noida, India, focuses on rehabilitating acid attack survivors by offering health, legal and economic support. Their landmark initiative, Sheroes Hangout Café, is managed entirely by survivors, providing them with employment opportunities. Four of the study participants were employees in the café at the time of data collection. Since most survivors face unacceptance in their homes and communities, the Chhanv Foundation offers accommodation through their facility, Sheroes Home. All study participants were availing this accommodation during the time of data collection.

### Participants and recruitment

Homogeneous purposive sampling^23^ was used to recruit study participants. Eight girls and women, aged 14–46 years, residing in Noida, India and who were survivors of acid violence, were selected for the study. All participants were unmarried (one was divorced) and came from a low-middle socioeconomic background. Most participants had not completed secondary education, though one held a master’s degree, and several were pursuing informal education or vocational training opportunities provided by the NGO.

Due to confidentiality and the public visibility of some survivors on social media platforms, detailed information such as time since attack and specific clinical characteristics has not been presented to avoid risk of identification. However, all participants had sustained severe acid burns, primarily involving the face, head, neck, arms, and torso, and had undergone multiple reconstructive procedures.

This study was part of a larger research project on stigma and discrimination faced by burn survivors in healthcare settings.^24^ The body mapping workshops were initially intended to explore healthcare-related stigma; however, participants’ discussions and creative expressions extended beyond this, highlighting experiences of social stigma, self-perception, and resilience. Because all participants took part in the group sessions, recruitment was not iterative, and theoretical saturation was not an intended goal. The Chhanv Foundation facilitated the recruitment process. We approached all survivors residing at the NGO facility during the study period who met the inclusion criteria and were available for the sessions. As the sample was determined through this context, it did not expand in cycles. None of those approached refused participation and there were no dropouts.

### Procedure

Body mapping, an arts-based, participatory method, was used to ensure comprehensive documentation of participants’ experiences. It is an intensive process requiring a high degree of reflexivity through art and subsequent discussions. The facilitator’s guide by Soloman J. titled ‘Living with X’ was used to preplan the sessions.^25^ To ensure participants’ comfort and allow sufficient time for reflection, four body mapping sessions, each lasting 3–6 hours, were conducted over 5 weeks. FGDs were conducted at the end of the last two sessions. All body mapping and FGDs were facilitated by PS and RR, hereafter referred to as ‘facilitators.’

In the first session, we outlined the purpose and procedure of the body mapping activity. Detailed participant information sheets were circulated and discussed. As some of the participants could not read, all the information was read out and their queries were addressed. Next, written informed consent was obtained from participants ensuring their voluntary participation. Participants were encouraged to ask questions and to think about what they would like to put on their body map. To initiate the body mapping process, participants lay down on a large piece of paper in a position of their choice. With assistance from the facilitators or another participant, an outline of each participant’s body was drawn onto the paper, serving as the foundation for subsequent sessions.

Our next three sessions began with participants being reminded of the voluntary nature of their participation. After that, they filled out their body maps based on prompts from the facilitators around themes such as personal and social experiences of stigma, shifting identity, memories of strength and vulnerability, and coping mechanisms. Participants had the freedom to discuss their thought processes, ask questions or seek clarification from the facilitators throughout the activity. Brief breaks were taken every 1.5–2 hours. Frequent informal one-on-one conversations between the facilitators and participants were adopted to interpret the symbols and visuals drawn on the body maps. These conversations were recorded as handwritten field notes by PS. Participants were provided with all necessary art supplies, including newspapers, paints, marker pens, coloured pencils and paint brushes. They were also encouraged to bring personal items, such as unidentifiable pictures, stickers and dry foliage to display on their map.

Two FGDs were conducted at the end of the third and fourth sessions to discuss the impact of acid violence on the participants’ lives through the lens of stigma. The FGD guide was translated into Hindi for ease of use. The discussions covered experiences in hospitals and other public spaces, instances of stigmatisation and discrimination, relationships with oneself and families, and coping strategies. Verbal conversations took place in both Hindi and English, with text written on the body maps in mostly Hindi. FGDs lasted between 75 and 90 min and were recorded, with detailed handwritten notes taken during the process. After completion, recordings were labelled with identification numbers, transferred to a secure drive location on PS’s laptop, and then deleted from the phone. The final body maps were returned to the respective participants.

Online supplemental file 1 includes an overview of each body mapping session (including prompts) and the FGD guide.

### Analysis

Analysis was a two-component process involving the body maps and FGDs as suggested by Gastaldo *et al*.^26^ First, the visual analysis of body maps began alongside data collection, as facilitators discussed individually or in small groups with participants to interpret and make sense of the symbols and visuals drawn on their maps. Once the activities were completed, high-resolution pictures were taken of all body maps. PS, proficient in both Hindi and English, familiarised herself with the content on the maps by reviewing them in detail. This was done ‘not to psychologically evaluate the participants through their art, but to gain insight into certain aspects of their logic, aspirations, desires, material circumstances and ways of handling particular issues’.^25^ Each body map was examined across dimensions of symbolism (eg, recurring motifs like hearts or scars), spatial arrangement (eg, what was highlighted or obscured on the body and positioning of elements) and textual annotation (written words and placement of text). Notes were made on these features and linked to corresponding FGD excerpts. This process allowed for systematic interpretation of both visual and verbal narratives as interconnected data sources. The body mapping images were uploaded to NVivo software for coding.

Next, PS listened to the FGD recordings to become familiar with the data, with attention paid to the tone and emotions of the participants. These recordings were then translated and transcribed into English by certified translators experienced with qualitative research. PS reviewed the transcripts against the original recordings to verify translation accuracy and contextual nuance. Final transcripts, along with field notes, were then uploaded to NVivo software for coding and analysis after removing any identifiable information.^27^

PS conducted the initial coding using an inductive approach to capture all relevant information, including the placement of symbols, patterns and text. Regular discussions between PS and VRK were held to minimise bias in coding. A preliminary codebook was developed, combining emergent themes from the body maps and FGDs. Codes were then clustered into categories reflecting stigma manifestations and coping mechanisms. Consensus was received through iterative discussions until agreement on interpretation was achieved.

Integration between the body maps and FGD data occurred through triangulation: patterns identified visually, for example, recurring stick figures representing relationships, were compared with FGD narratives on friendships, family and social acceptance. This integration ensured that the visual data illustrated the findings and actively contributed to theme development. Further discussions with coauthor JJ assisted in refining interpretation and resolving any discrepancies. During analysis, we assessed data saturation, as the point at which additional review of the data did not yield new codes, meanings or patterns relevant to the study objectives. Through repeated examination of the body maps, field notes and FGD transcripts, we observed that no new insights were emerging, and therefore analytic (data) saturation was considered reached within the dataset generated.

Themes were developed by clustering codes and categories based on their relevance to the respondents’ experiences and perceptions. As the analysis progressed, some initial codes were discarded if they did not align with the emerging structure. Through detailed deliberation and discussion among the authors, a final list of main themes and subthemes was established. The study adheres to the consolidated criteria for reporting qualitative research (COREQ), and a completed checklist is included as online supplemental file 2.

### Ethics

We maintained participant privacy by conducting all activities in a previously booked hall where only the facilitators and participants were present. The space included a carpeted floor and some seating sofas. We often engaged in non-study-related conversations because participants expressed interest, sharing information about our roles, research, and backgrounds. Each participant selected a pseudonym for use in the study reports and on the body maps. Photos taken during the process were approved by the participants. They were compensated for travel expenses and lunch and refreshments were provided throughout the study. We had initially planned for a INR750 (≈US$8.5) incentive for each participant; however, the NGO authorities advised against it as it could have created differences between those who participated and those who did not. Instead, we provided the same amount per participant to the NGO for overall survivor skill-building and well-being.

At the time of data collection, two participants were minors: Radhika, aged 16, and Molly, aged 14. Written assents were obtained from both. During the study, they were residing in the NGO-provided accommodation. Radhika’s mother, also an acid attack survivor, provided consent for her participation, with facilitation from the NGO. In Molly’s case, direct parental consent was not feasible, as her father was the perpetrator of her attack and was in prison. Her mother resided in a remote village, and she lacked the economic means to provide rehabilitative care. On Molly’s arrival at the NGO, her mother had signed a general consent form authorising the NGO to oversee Molly’s participation in research studies and other activities, including determining her consent. Before including Molly in this study, PS discussed with an NGO representative regarding her involvement. The representative supported her participation, noting that Molly was fond of art and suggested that the body mapping activity might be beneficial for her. However, they advised that Molly might not feel comfortable discussing her attack and experiences in a group setting, which we respected. She preferred to discuss her body map directly with PS, necessitating more one-on-one interaction compared with the other participants.

Given the sensitive nature of the study, we monitored participants for signs of distress throughout all sessions. Our collaboration agreement with the NGO included provisions for immediate referral to them for any psychosocial support needs in case of significant emotional distress. In addition, contact details for free counselling through mental health helplines were shared along with the consent forms. We also conducted informal debriefings before and after each session to allow participants to process their experiences and ensure emotional well-being. No identifiable information was retained from participants, and we advised them not to put any sensitive information on their body maps. Facilitators were also trained in ethical data collection.^28^

### Reflexivity statement

We were an interdisciplinary team comprising three public health researchers (two women and one man) and one social worker (woman). None of us had prior engagement with the NGO or participants.

PS, trained in qualitative research, and RR, experienced in body mapping and work with survivors of violence, facilitated all field activities. Although we shared some aspects of identity with participants (such as gender), we recognise that our positionality was shaped by forms of privilege associated with education, caste, social class, and professional status. These intersecting differences created uneven power dynamics that may have influenced how participants perceived us, what they chose to share and how comfortable they felt expressing distress or critique. At the same time, shared experiences of navigating patriarchal norms as women allowed us moments of relatability and empathy. However, we remained conscious that our own experiences of patriarchy were fundamentally different from the extreme gendered violence experienced by survivors. This dynamic of being partially relatable yet fundamentally ‘outsiders’ to their lived realities required ongoing reflexivity. We acknowledge that despite our intentions, we could only attempt to ensure that interpretations remained grounded in participants’ perspectives rather than filtered through our own assumptions or professional lenses.

To minimise power imbalances, we used participant-led discussions, flexible facilitation and encouraged survivors to set the pace and depth of sharing, particularly during emotionally sensitive activities such as drawing scars or recounting experiences of stigma. We also reflected regularly on how our presence, reactions and emotional responses might shape the space. The broader author team—JJ, an international injury epidemiologist, and VRK, a medical doctor with expertise in burns care and research—contributed to conceptual framing, iterative analysis, and interpretation. Regular team discussions provided reflexive spaces to examine our assumptions and positional influences to better understand how our identities may have shaped this study.

## Results

Body maps were developed for all eight participants. Through thematic analysis of data originating from the body maps and FGDs, we identified two key themes: ‘Factors contributing to stigma’ and ‘Factors mitigating stigma.’ Contributing factors include three sub-themes: devaluation of bodies with disfigurement, familial unacceptance and abuse, and opportunity loss. Factors mitigating factors include two subthemes: self-acceptance and support of NGO.

### Factors contributing to stigma

#### *‘With my burnt face, I will never be happy again’* - Devaluation of bodies with disfigurement

This subtheme denotes the social stigma faced by survivors due to their disfigurement. Participants reported encountering poor public perceptions and negative societal attitudes, which significantly impacted their social interactions and sense of belonging. They felt that these strained relationships and negative societal perceptions were largely due to their disfigured appearance. The fear of public scrutiny and the pressure to conform to societal norms of beauty led to an increased consciousness about their appearance, resulting in feelings of inadequacy. This scrutiny extended to romantic relationships, with participants expressing anxiety about potential rejection due to their scars. The societal emphasis on physical appearance made it difficult for participants to envision a future where they could find a partner who ‘accepted them as they were’. Further, as most of them were under 30 years old (within what is socially regarded as the ‘marriageable age’), concerns around physical appearance, social acceptance and marriage prospects were central to their narratives. Family members also often echoed this concern, adding pressure related to marriage and societal acceptance.

*Everyone’s behaviours used to make me feel as if I was being mentally tortured. It started bothering me a lot. Even my husband who used to love me started being very rude, saying things like ‘no one would even urinate on your ugly face*”- Angel

*My relatives kept telling me that I was so beautiful but now with my burnt face, I will never be happy again. I could have gotten married and had a family, but no one will look twice at me now. It was very hurtful*.- Paru

As a result of facing poor societal attitudes, participants mentioned a severe loss of self-confidence following their burns. One participant, Angel, recounted that she could not look at a mirror for almost 3 years after her attack, mentioning that her new appearance led to a drastic change in perception of self-worth. All participants reported a severe psychological toll of burns, with some participants, such as Paru, mentioning a lack of desire to live and considering death as a preferable option to the life they had post-attack.

*It was a very bad time for me… I did not see any point in living and felt like I should die only because my reality was so very painful*. – Paru

*Before I always used to be depressed, and I could never look at myself in the mirror. I used to feel that my life had ended because who could stay with someone like me.* – Nayab

**Figure 1:**
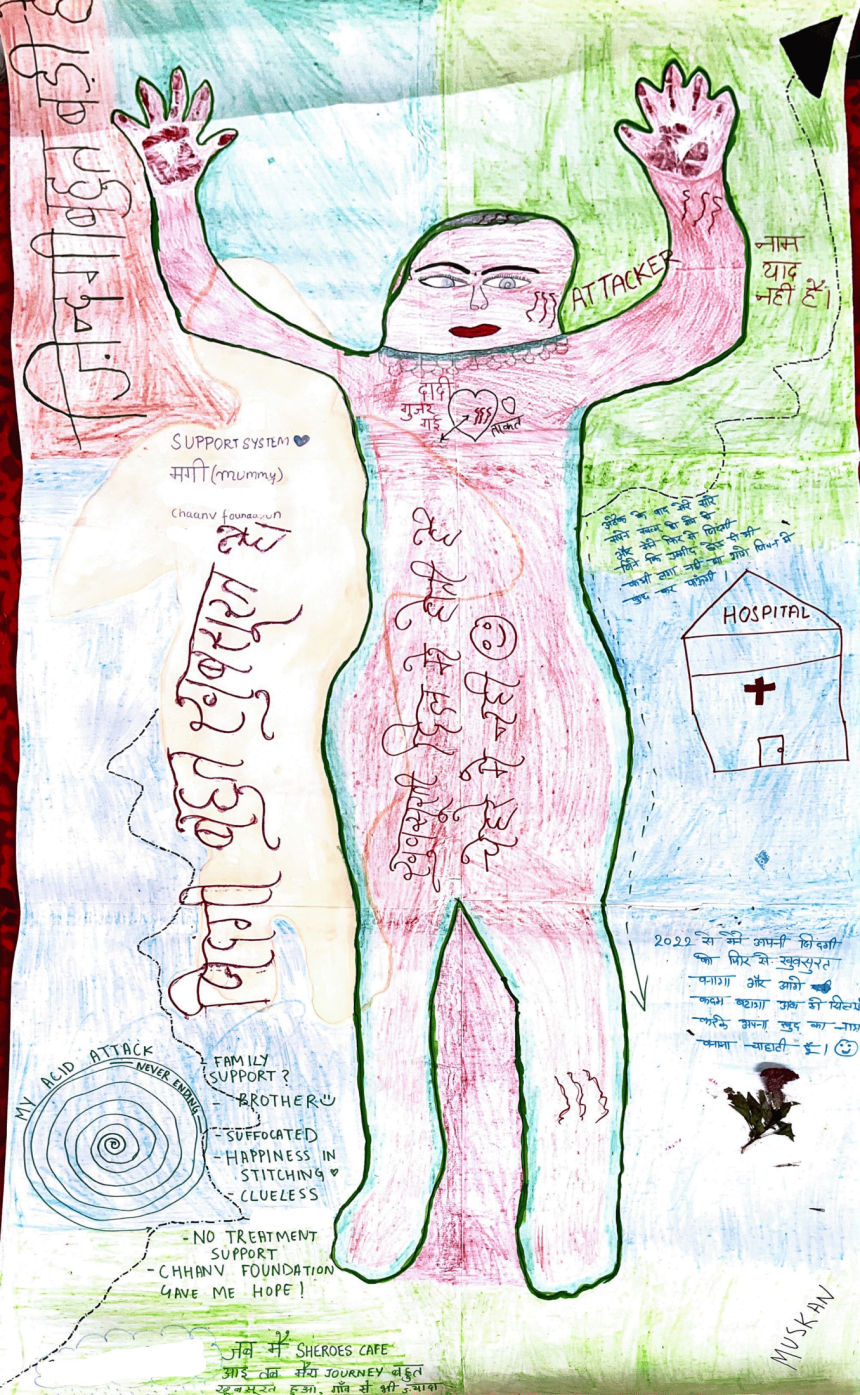
Muskan’s body map.

Muskan’s body map, which says “*After my attack, I had lost all my dreams and lost the will to live. I didn’t think I could achieve anything in life with a face like mine*” (figure 1).

#### *‘Mix poison in it and give it to her, she will not know’* - Familial unacceptance and abuse

This subtheme reports challenges of familial unacceptance and abuse faced during participants’ recovery from the acid attack. They shared that families often struggled to cope with the visible scars and disfigurement, leading to a strained dynamic where survivors felt isolated and stigmatised within their own homes. This frequently manifested in abusive behaviour, with family members directing their frustration towards the survivor. Participants reported being asked to cover their faces at all times, especially when leaving the house or meeting people. They also faced verbal abuse from their families due to their scars. In the case of two participants—Komal and Paru—families attempted to end the survivor’s life, while they were recovering. The family considered death to be a kinder outcome to living with the stigma associated with burns.

*My father’s behaviour became bad as he started verbally and physically abusing me. My aunt used to say, ‘Who will keep such girl at home, we don’t want this big burden on our head.-* Molly

*My uncle asked my brother what food I eat, to which he responded that I couldn’t and that I only drink juice at the moment as I was on a liquid diet. My uncle said, ‘then take the tender coconut water, mix poison in it and give it to her, she will not know*.’- Komal

Participants reported low emotional and caregiving support as they felt that women survivors face unique challenges. Traditionally seen as the caregivers, they reported finding themselves in a vulnerable position where there was no one to take care of them during their recovery. This lack of support was particularly pronounced in households with other dependents, such as children or elderly family members, as caregiving efforts were often redirected to them.

Further, women are often perceived as bearers of the family’s dignity in Indian culture. The attack and the resultant disfigurement were viewed by family members as ‘spoiling’ this dignity, leading to feelings of shame for the participant. In addition, participants mentioned that family members often excluded them from social activities postburns due to fear of being (mis)judged or ill-treated in public spaces. This led to participants having limited mobility, effectively confining them to their homes, increasing their feelings of loneliness and anxiety.

*When I was admitted, my mother was in the hospital taking care of me, but my aunts kept pressurising her to go back to the village as she had household responsibilities and other children to care for. They told her to leave me alone in the hospital. My mother used to cry a lot*. – Komal

*Whenever I go home, I get tied up in one room and don't get to come out of the house… My family members don’t allow me to come out of the house. They say that if I go outside, the world will see me, and they would talk badly about me*.- Muskan

**Figure 2:**
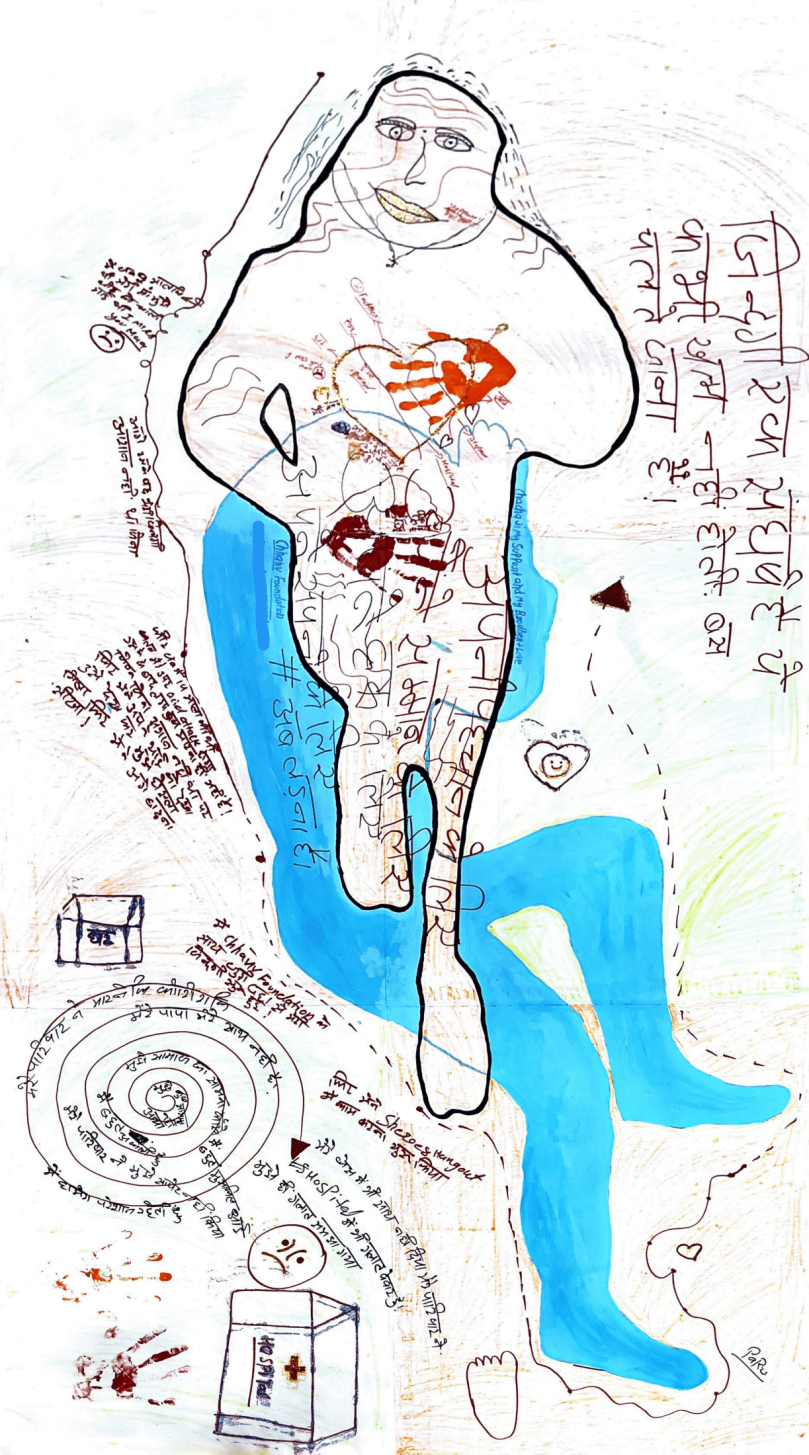
Paru’s body map.

Paru’s body map, which says “*I faced a lot of difficulties while going out into the public… My family did not support me with my legal case of acid attack*” (figure 2).

#### *‘You cannot do anything….’* - Opportunity loss

This subtheme reports on the barriers to economic and educational opportunities encountered by survivors. Participants highlighted how even after recovery, their disfigurement continued to impede their access to educational and job opportunities.

Opportunity discrimination was a critical aspect of stigma. Participants shared that after the acid attack, interruptions in their education and employment due to medical treatment left them financially dependent on their families, further straining household dynamics. However, even after recovering from the acute phase, when they sought employment opportunities, they were often turned away due to their appearance. For survivors who were still in school or university, their institutions were reluctant to re-admit them, fearing that their presence might disturb other students or attract negative attention. This denial of employment and educational opportunities limited the survivors’ ability to achieve economic independence, marginalising them within society.

One participant noted that in Indian mythology, scars are often associated with evil, as demons are depicted with scars. She believed that this cultural belief led to survivors being perceived as ‘cursed’ by potential employers, due to which their candidatures were rejected. Participants felt that such ingrained cultural biases further marginalised survivors, minimising their ability to pursue future opportunities.

*If you go for job in any place, they will reject you at once because of your face. You cannot do anything…. Now in this café where we are working, even now some people will see our face and go away from the restaurant*.- Nayab

**Figure 3:**
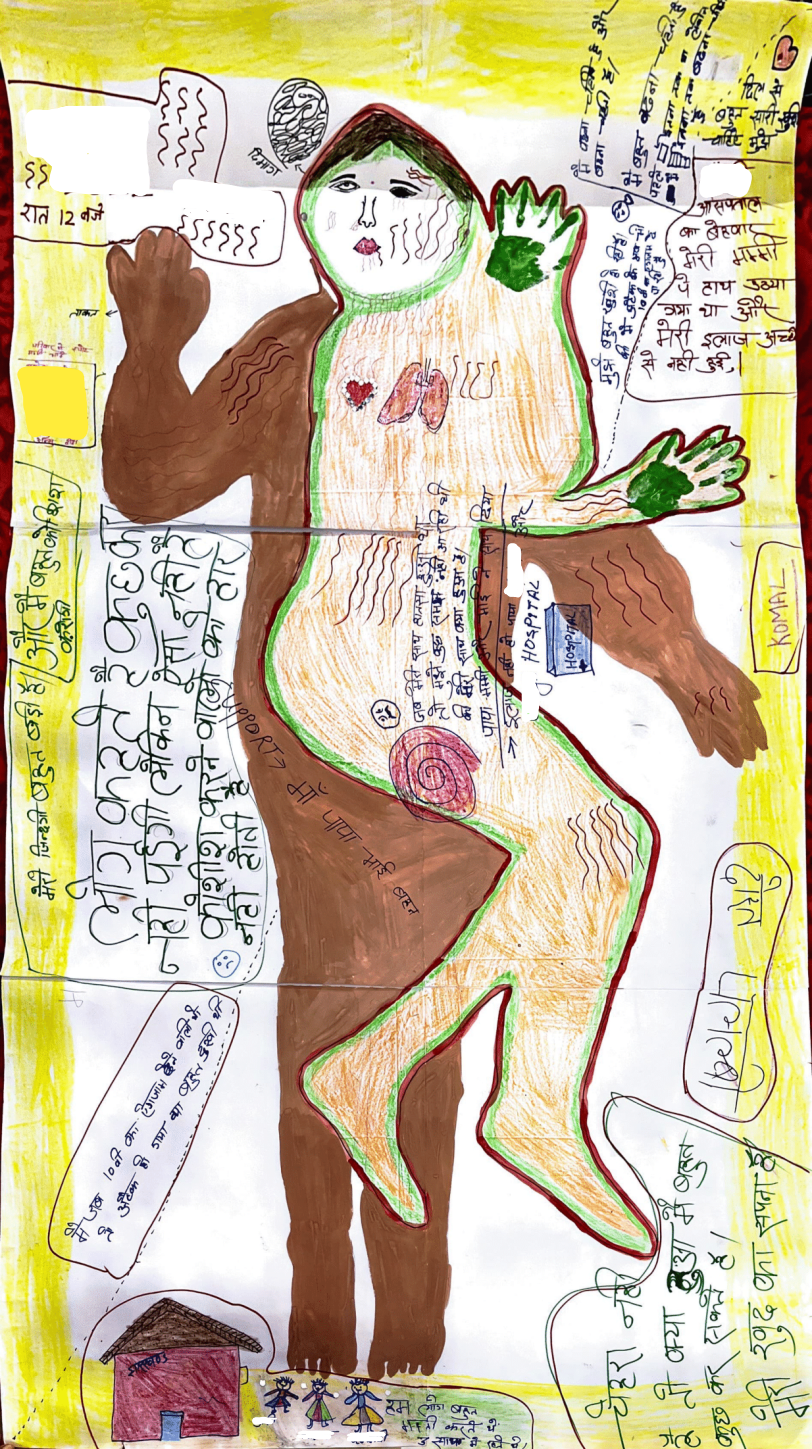
Komal’s body map.

Komal’s body map, who was 15 years old at the time of attack and faced a disruption in continuing her education (figure 3).

### Protective factors mitigating stigma

#### *‘Life is big, life is beautiful!’* - Self-acceptance

This subtheme reports the processes and strategies that participants used to rebuild their self-confidence and esteem through adopting a positive mindset and peer support.

Participants described counselling themselves through positive self-talk and reflection, gradually rebuilding their self-esteem and confidence. This inner strength enabled them to stand up for themselves within their families and extended social circles. Participants highlighted that for most of them, self-acceptance was a critical step towards overcoming the psychological trauma of their disfigurement and moving forward with their lives. Many survivors adopted a positive mindset, believing that whatever happened was for the best as the acid attack allowed them to leave their villages and gain economic independence and freedom that they could not have gotten otherwise.

*Those who keep trying, never loose in life and I want to always keep trying.-* Radhika

*I have learnt to accept myself by saying ‘it is okay. It is not my mistake. Whoever has done it, it is their mistake. But whatever life I have got now, I have to live it.’ It is as they say that if God gives you pain, he will also give you the strength to bear the pain. It is okay*. – Pari

*Life is big, life is beautiful!* - Muskan

Further, participants also expressed the importance of finding their community of survivors in dealing with stigma. Sharing their stories and experiences with others who had faced similar challenges created a sense of togetherness and understanding. They reported that this network of support was critical in helping them feel less isolated and more empowered, as they were now able to share coping strategies, celebrate victories and offer encouragement and hope to each other.

*I am seeing [survivors] living lives their way. Without covering their face, they are going wherever they want. So now I also have courage to roam around… If I had not met them, then I never would have been able to do all of this*. - Komal

*I am more than my scars and being with [survivors] has made me fall back in love with myself*. - Angel

While all participants described some form of self-acceptance, the process and pace varied considerably. Those who had lived with their injuries for several years generally mentioned higher levels of confidence and self-compassion, often attributing this to their prolonged engagement with the NGO and peer networks. In contrast, survivors who were more recently attacked or younger in age expressed ongoing struggles with body image and public visibility, suggesting that self-acceptance can be a gradual, non-linear process shaped by both time since injury and the availability of sustained social support.

**Figure 4:**
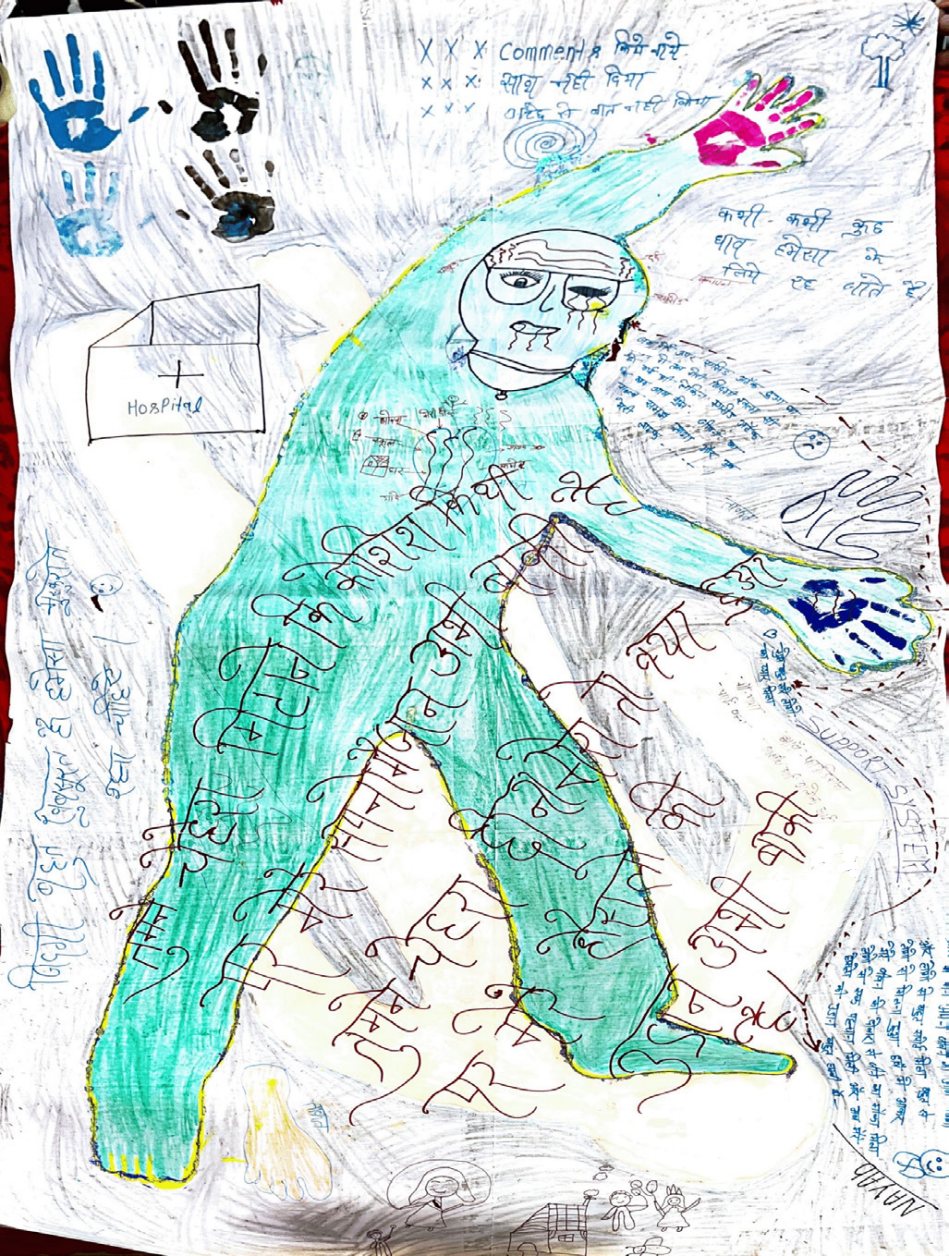
Nayab’s body map.

Nayab’s body map, which says “*You tried to erase my face, but my dreams still have life. So, what if you stole my face from me, my courage still remains*” (figure 4).

#### ‘*Through the NGO, everything has been better’* - Support of NGO

This subtheme reports the significant role played by the NGO in supporting the acid attack survivors through various means, including providing access to opportunities, promoting financial independence, and empowering themthrough advocacy and awareness-raising.

All participants repeatedly expressed that the NGO played a critical role in mitigating the social stigma faced by survivors. It provided survivors with access to essential services such as medical treatment, legal guidance, and educational opportunities. Participants felt that these resources were key in helping them navigate their lives postattack and reintegrate into society. Further, they mentioned that the NGO facilitated financial independence by offering skill-building and vocational training, which helped improve survivors’ employability.

Participants also reported that engaging in advocacy efforts and raising awareness about acid violence through the NGO allowed them to convert their trauma into a source of empowerment. By participating in campaigns and supporting other survivors, participants found a sense of purpose and resilience. This involvement not only helped them cope with stigma but also contribute to broader societal change by educating the public and advocating for stronger legal protections and support systems for acid attack survivors.

*Chhanv is everything to me and my mother [who is also an acid attack survivor]…. I don’t know where we would have been without it.-* Radhika

*When I was on my own, nobody took me… But now through the NGO, everything has been better. Now we directly speak to doctors and lawyers, and they actually listen to us!…. So many things have changed since 2013 and it all our hard work through the NGO as we have been working tirelessly advocate for our rights.*- Paru

**Figure 5:**
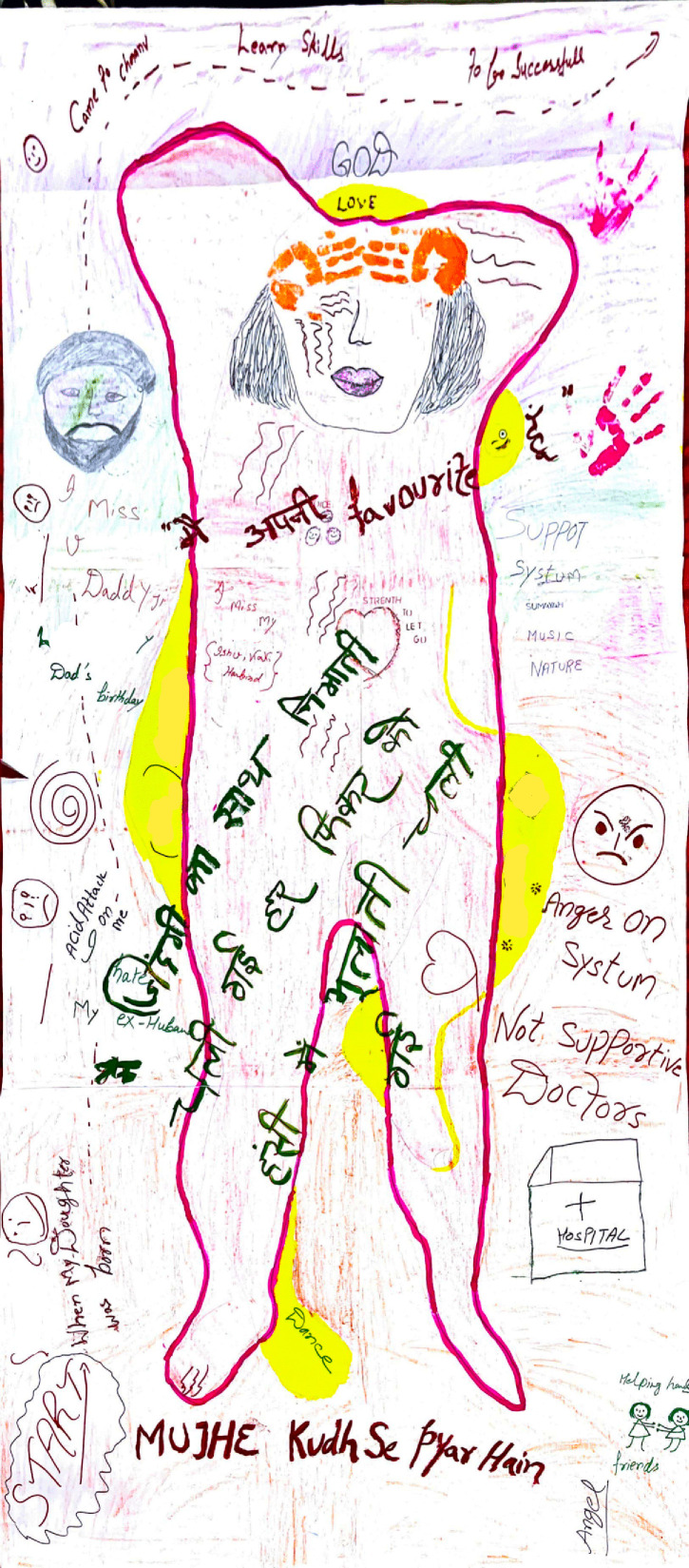
Angel’s body map.

Angel’s body map, which says “*Come to Chhanv… Learn skills… To be successful*” (figure 5).

## Discussion

We triangulated two qualitative methods to document acid attack survivors’ social stigma in Noida, India. First, by incorporating body mapping, we provided a platform for survivors to articulate their experiences in a multidimensional and participatory manner. Second, the subsequent FGDs with participants helped in deeper understanding of perceived underlying reasons for such stigma. The findings highlight that stigma is shaped by contributing factors such as familial rejection, societal devaluation and opportunity loss, while mitigating factors like NGO support and self-acceptance play a critical role in resilience and recovery. Existing evidence largely deals with the legal aspects of acid violence,^419^,^21^ with limited research addressing the social stigma and exclusion of acid burn survivors in India. Our findings aimed to fill this knowledge gap in understanding the challenges of acid attack survivors in India.

This study highlights the social impact of acid violence. Similar to facial disfigurement arising from conditions such as cancer, participants experienced devaluation and isolation, a phenomenon supported by global evidence highlighting how societal attitudes towards physical appearance can perpetuate marginalisation and negative stereotypes.^1729^,^31^ The relevance of these findings extends beyond the Indian context as research from other LMICs reports survivors experiencing social exclusion, diminished self-esteem, and barriers to economic and social opportunities.^232^,^35^ The present study also illustrates how cultural biases, such as the association of scars with ‘evil’ or demons in Indian mythology, shape societal attitudes towards survivors. This is not unique to Indian culture as it has also been noted in wider South Asian belief where disfigured individuals have been portrayed as evil and fearful.^36^ By evidencing these patterns, our study contributes to the growing literature on the intersection of cultural norms, disfigurement, and stigmatisation.

Research on burn survivors overall also echoes many of our findings, documenting persistent stigma, social exclusion and psychological distress, particularly in the case of facial burns.^37 38^ However, for acid violence survivors, this stigma is often compounded by moral judgement and gendered blame, given the intentional nature of the attack. While disfigurement-related stigma may cut across different burn aetiologies, acid violence can indicate a distinct intersection of appearance stigma, along with gender-based violence, further deepening survivors’ marginalisation.

Acid attacks are deeply rooted in patriarchal structures that view women as subordinate and often seek to control their autonomy, with perpetrators wanting to enforce their masculinity and power.^39 40^ An important dimension of the narratives was the evolution of survivors’ self-perception and empowerment from the point of the attack to the study’s time frame, years later. Initially, participants described feelings of shame, worthlessness, and isolation. However, through collective engagement with other survivors, many reported a shift towards reclaiming their identities, finding purpose, and increased autonomy. This temporal shift also reflected changes in the nature of stigma itself—from acute experiences of rejection and self-blame in the immediate aftermath to subtler, long-term exclusions such as social avoidance and constrained opportunities, highlighting that stigma is not static but evolves in tandem with survivors’ social reintegration and recovery. This process of positive healing was facilitated by sharing their stories, advocating for others, and running the Sheroes Hangout Café. These findings align with theories on post-traumatic growth, which suggest that individuals can derive meaning and empowerment from their traumatic experiences, leading to improved relationships and a sense of personal strength.^41 42^

There is a pressing need for comprehensive rehabilitation programmes that go beyond medical treatment to include psychosocial health services, vocational training and legal assistance. Our findings highlight the transformative role of support structures, particularly the NGO’s support. However, formal support systems, including governmental initiatives, remain insufficient in addressing the multifaceted needs of survivors. In addition, strengthened legal measures addressing acid violence are needed. In India, acid attacks are criminalised under Sections 326A and 326B of the Indian Penal Code, with penalties including imprisonment and fines.^43^ The Supreme Court has also mandated restrictions on acid sales and a minimum compensation of 3 lakh INR for survivors.^44^ However, massive gaps continue to remain in enforcement, with inconsistent regulation of acid sales, delays in compensation, and negligible access to rehabilitation services.^21 45 46^ In practice, this could involve designated one-stop crisis and support centres for survivors, integrating long-term vocational support into survivor welfare schemes, and stronger local oversight to enforce compensation and acid regulation. Further, promoting women empowerment and dismantling patriarchal structures through evidence-driven measures can help reduce overall violence against women. One such comprehensive approach is the RESPECT Women framework by the WHO which includes strategies such as creating safe environments, reducing poverty, and preventing abuse.^47^

We found body mapping to be an effective tool for data collection, offering a unique approach to understanding and representing people’s experiences. Arts-based methodologies have evolved from the traditional ways of doing research with the understanding that people’s lives and experiences are multifaceted and unlikely to be captured comprehensively through only intellectual responses.^25 26^ The ‘story’ that comes out through body mapping enables a high degree of reflexivity, not only for researchers, but also for participants, resulting in outputs that are unlikely to have been produced through traditional quantitative or qualitative means. Since our study was potentially emotionally distressing, we found this approach to allow for the expression of frustration and pain that may be challenging to articulate verbally. This was also helpful as literacy levels differed between participants, with only some knowing how to write in either Hindi or English. Further, as noted by Skop,^48^ this act of storytelling, combined with the visual representation in body mapping, was found to contribute to a sense of empowerment and agency for participants, helping them reclaim their narratives.

To our knowledge, this is the first study globally to use the body mapping methodology in the context of acid attack survivors and/or stigma. While the findings provided valuable insights into manifestations of social stigma, there are some limitations. First, we relied on self-reported data, which can be influenced by recall or social desirability bias, although we have no reason to believe that this would polarise responses or influence findings. Second, as the study was conducted with participants from a specific geographic location, the findings may not be generalisable to all acid attack survivors across India or other regions. However, our aim was largely exploratory and not representative, as we wanted to document narratives of stigma in-depth. Finally, though the body mapping methodology can be highly effective for engaging participants, it is inherently subjective and there may be loss of nuance between participants’ artwork and researcher interpretation. To minimise this, we undertook extensive field notes while engaging in one-on-one conversations with the participant and larger FGDs which allowed us to triangulate and validate the data.

## Conclusions

This study provides a novel contribution to understanding the lived experiences of stigma experienced by acid attack survivors in India. Our findings call for a more holistic approach to survivor rehabilitation, including psychosocial support, vocational training and legal protection. Addressing gendered violence requires strict enforcement of existing laws on acid sales, survivor compensation, and rehabilitation. By amplifying survivor voices and advocating for evidence-based interventions, we can move towards a more inclusive and equitable society where those impacted by violence can reclaim their identities and thrive.

## Supplementary material

10.1136/bmjph-2025-002693 online supplemental file 1

10.1136/bmjph-2025-002693 online supplemental file 2

## Data Availability

No data are available.
