# Supplementary material for: Stigma, disfigurement and resilience among acid attack survivors: a qualitative body mapping study in Noida, India
Source: BMJ Public Health. 2026 Jan 27;4(1):e002693. doi: 10.1136/bmjph-2025-002693 (PMC12853538; doi:10.1136/bmjph-2025-002693)
Supplement: online supplemental file 1 [file bmjph-4-1-s001.docx]

**Supplementary 1**

# Body Mapping Session Plan

## Session 1

**Objectives:** Introduce participants to the study purpose and the body mapping method; establish trust, safety, and voluntary participation; initiate creation of the map.

**Activities & Prompts:**

- Ice-breaking activities
- Discuss study objectives, circulate and explain participant information sheets and consent forms
- Discuss ground rules around confidentiality, respect, and voluntary sharing for all sessions.
- Creating the body outline: Each participant traced their body in a chosen posture.
  *Prompt:* “How would you like to represent yourself today?”
- Support Figure: Outline a support figure as a ‘backbone’ to the main outline to represent people or things providing emotional or practical support.
  *Prompt:* “How would you like to represent your support figure today?”
- Group reflection

## Session 2

**Objectives:** Facilitate exploration of personal and social experiences of disfigurement, shifting identity, and societal perceptions; identify memories of strength and vulnerability.

**Activities & Prompts:**

- Ground rules
- Power points: Identify and highlight areas of the body associated with personal strength or resilience.
  *Prompt:* “Where in your body do you feel power or courage?”
- Personal symbols: Create symbols of identity, hope, or faith.
  *Prompt:* “If you could draw a symbol that represents your journey or identity, what would it be?”
- Marks and scars: Drawing of scars.
  *Prompt:* “What marks (visible or invisible) tell your story?”
- Body image and identity: Reflect on changes in appearance, self-worth, and emotions after the attack.
  *Prompt:* “When you think about yourself after the attack, how and what do you feel?”
- Public perceptions and relationships: Explore societal reactions and changes in interactions in public spaces.
  *Prompt*: “How did people’s behaviours toward you after the attack?” “What happens when you go out in public spaces such as schools, parks, or restaurants?”
- Group Reflection.

## Session 3

**Objectives:** Explore experiences of stigma – particularly in healthcare spaces – and suggestions on what could be done differently.

**Activities & Prompts:**

- Ground rules
- Life paths: Reflection on personal journeys and future aspirations.

*Prompt:* “Where did you come from and where do you want to go? Draw a line around your body map marking key life points.”

- Internal, perceived, and enacted stigma: Participants identified and drew instances of stigma in healthcare, community, or family spaces.

*Prompt*: “What does stigma mean to you? Where and how do you experience it?”

- Hospitals and healthcare: Reflect on experience while visiting seeking healthcare and interactions with doctors, nurses, or other patients.
  *Prompt:* “What did you feel before, during, and after visiting hospitals?” “How did this differ from when you sought care before your acid attack?” “How did you experience stigma in these instances?”
- Respectful care: Reimagination of healthcare to be stigma-free and inclusive.

Prompt: “How can hospitals support you better?” “How would you like hospital staff to treat you?” “What would make your experience more positive?”

- Group reflection
- Structured FGD

## Session 4

**Objectives:** Focus on recovery, resilience, and collective strength in coping with and mitigating stigma.

**Activities & Prompts:**

- Ground rules
- Recovery and coping: Discuss emotional healing, self-care practices, and sources of support.
  *Prompt:* “Who or what helped you heal after your attack?” “What do you do when you want to feel better?” “How has being part of this community or NGO impacted your life?”
- Suggestions: Encourage participants to express hopes for broader systemic or societal change.
  Prompt: “What do you wish could be better for survivors like you?”
- Slogan creation: Each participant writes a personal slogan symbolizing hope, identity, or advocacy.
  *Prompt:* “If you could tell the world one message about yourself, what would it be?”
- Independent completion: Final touches of embellishing the maps using paints, mirrors, glitter, or other symbolic materials.
- Group reflection
- Structured FGD

# Focus Group Discussion Guides

## FGD 1 (after session 3)

**Theme: Experiences of Stigma and Discrimination**

Introduction

- Revisit purpose of the study
- Reaffirm voluntary participation and emotional safety
- Reiterate ground rules

Understanding Stigma

- When you hear the word stigma, what does it mean to you?
- In your experience, when do you feel that people treat you differently because of your burns or scars?
- Can you describe how this makes you feel or what thoughts come up in those moments?

Experiences in Public and Community Spaces

- How do people in your community or neighbourhood react when they see you?
- What happens when you go out in public – for example to markets or see a movie or to a restaurant, etc?
- Do you ever change your behaviour (like covering your face, avoiding places, etc.) because of how others react?
- What kinds of comments or looks have stayed with you?

Family and Relationships

- How did your family members, relatives, and friends react after the attack?
- Has your relationship with them changed since then?
- How do (or don’t) they support you now?

Experiences in Healthcare

- Can you tell me about your experiences when you seek healthcare in hospital facilities?
- How were you treated by doctors, nurses, or other patients?
- Was there any time you felt judged or disrespected?

Suggestions for hospitals

- What kind of support or behaviour made you feel safe and respected?
- What would make hospitals more comfortable for people like you?

Closing Reflection

- What would you want people to understand about living with burns or scars?
- If you could change one thing about how society treats survivors, what would it be?
- Emotional debrief

## FGD 2 (after session 4)

**Theme: Recovery, Resilience, and Self-Acceptance**

Introduction

- Revisit purpose of the study
- Reaffirm voluntary participation and emotional safety
- Reiterate ground rules

Coping and Emotional Recovery

- What helped you heal emotionally after the attack?
- Who or what gives you courage when you feel low?
- Are there things you do — like art, music, talking to friends — that make you feel better?

Role of the NGO and Peer Support

- How has being part of this NGO or this group changed your life?
- Have you found support or friendship among other survivors?
- Are there things that NGOs or the government could do better?

Self-Image and Identity

- How do you see yourself today compared to just after the attack?
- What does confidence or self-acceptance look like for you now?
- How do you deal with days when you don’t feel strong?

Hope and Advocacy

- What message did you write on your map about what you want to tell the world about yourself? Discuss its significance
- What changes do you want to see in how survivors are treated?
- What advice would you give to someone who has just gone through an acid attack?

Closing Reflection

- Is there anything you’d like to add or share that we haven’t discussed?
- What did it feel like to take part in these sessions?
- Emotional debrief

__________________
